# Data on differential multivariable risk prediction of appropriate shock vs. competing mortality

**DOI:** 10.1016/j.dib.2018.11.025

**Published:** 2018-11-09

**Authors:** Leonard Bergau, Rik Willems, David J. Sprenkeler, Thomas H. Fischer, Panayota Flevari, Gerd Hasenfuß, Dimitrios Katsaras, Aleksandra Kirova, Stephan E. Lehnart, Lars Lüthje, Christian Röver, Joachim Seegers, Samuel Sossalla, Albert Dunnink, Rajevaa Sritharan, Anton E. Tuinenburg, Bert Vandenberk, Marc A. Vos, Sofieke C. Wijers, Tim Friede, Markus Zabel

**Affiliations:** a University Medical Center Göttingen, Dept. of Cardiology and Pneumology, Göttingen, Germany; b University Hospital of Leuven, Leuven, Belgium; c University Medical Center Utrecht, Dept. of Medical Physiology, Utrecht, Netherlands; d University Medical Center Utrecht, Dept. of Cardiology, Utrecht, Netherlands; e Attikon University Hospital, Dept. of Cardiology, Athens, Greece; f DZHK (German Center for Cardiovascular Research), Partner Site Göttingen, Göttingen, Germany; g University Medical Center Göttingen, Dept. of Medical Statistics, Germany; h Division of Cardiology, Dept. of Internal Medicine II, University Hospital Regensburg, Regensburg, Germany

## Abstract

This data article features supplementary figures and tables related to the article “Differential Multivariable risk prediction of appropriate shock vs. competing mortality – a prospective cohort study to estimate benefits from implantable cardioverter defibrillator therapy” (Bergau et al., 2018) [1]. The figures show the clinical study CONSORT graph (data that show the number of patients not-analyzable as well as a distribution of patients by outcomes) and the correlation scatter plot for risk scores of appropriate shock vs. mortality (data that show the calculated score values of the two scores plotted against each other). The tables show the results for the univariate Cox regressions for prediction of mortality and appropriate shock. For further information, please see Bergau et al. (2018) [1].

## Specifications table

**Table:** 

Subject area	*Medicine*
More specific subject area	*Clinical study data*
Type of data	*Figures, tables, and text*
How data was acquired	*The data were acquired in a clinical study*
Data format	*Figures, tables, and text*
Experimental factors	*Observational clinical diagnostic study in ICD patients*
Experimental features	*CONSORT graph, correlation scatterplot of shock score vs. mortality score, calculation formulae for both scores, univariate Cox regression for both endpoints*
Data source location	*Göttingen/Germany; Leuven/Belgium; Utrecht/The Netherlands; Athens/Greece*
Data accessibility	Data is available in this article
Related research article	Bergau L, Willems R, Sprenkeler DJ, Fischer TH, Flevari P, Hasenfuss G, et al. Differential multivariable risk prediction of appropriate shock versus competing mortality – a prospective cohort study to estimate benefits from ICD therapy Int J Cardiol. 2018; 272:102–7 [1].

## Value of the data

• The CONSORT graph data of the prospective clinical study is shown, giving insight into the distribution of patient subjects in the study.
• The correlation scatter plot data for calculated risk score values of appropriate shock vs. calculated risk score value for mortality is shown, original pairs of score values can be discerned in the graphics and are shared as a file.
• The univariate Cox regression data for prediction of mortality and appropriate shock (unadjusted and adjusted for base model) are shown, giving insights into the basic statistical data before multivariate analyses.

## 1 Data

The data article features supplementary figures and tables related to [1]. See the abstract above for further details describing the data.

## 2 Experimental design, materials and methods

**Fig. 1:**
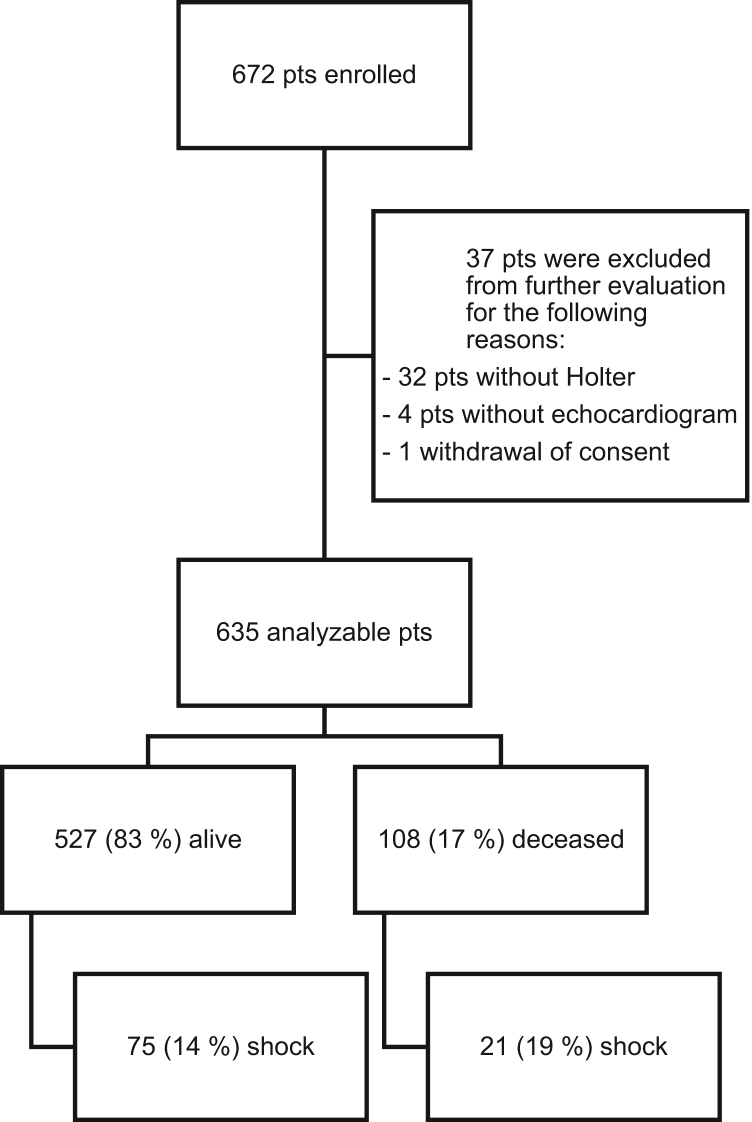
CONSORT graph for patient enrolment, patients not considered for final analysis and clinical endpoints.

**Fig. 2:**
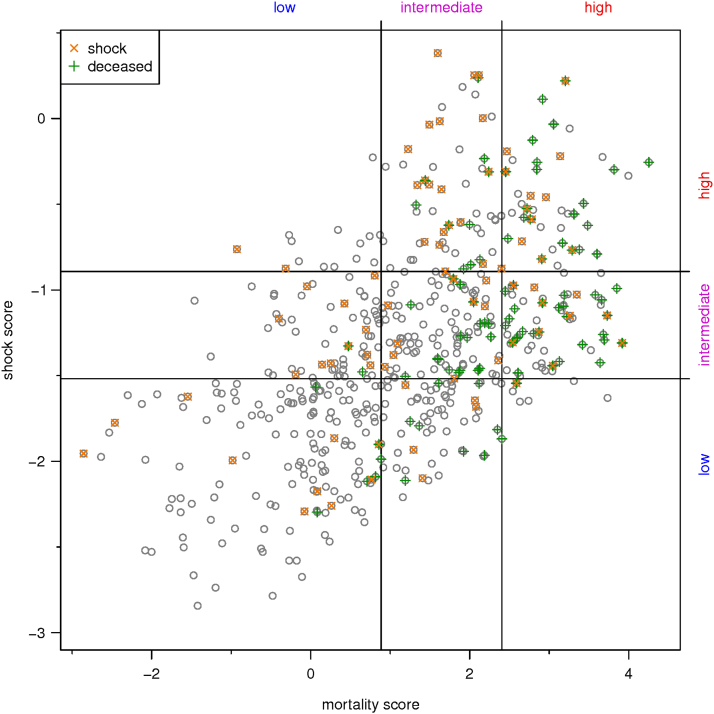
Correlation scatter plot for calculated risk score values of appropriate shock vs. calculated risk score value for mortality (*r* = 0.56, *p* < 0.001) Horizontal and vertical lines depict the low, intermediate, and high risk values of each score. The figure shows that the correlation is at best moderate despite mathematical significance. Thus, all-cause mortality risk does not coincide well with appropriate shock risk. Individually, a low risk of appropriate shock does occur with a high competing risk of death limiting the effectiveness of implantable cardioverter defibrillator therapy in a given patient (lower right quadrant). Vice versa, individual patients can be identified with fairly high risks of appropriate shock and concomitant moderate risks of death (upper left quadrant). These individuals are expected to have a higher life-prolonging effect of their implantable cardioverter defibrillator therapy, i.e. higher implantable cardioverter defibrillator benefit. The original score values are attached in a table (csv file).

A prospective international clinical study was initiated as part of the European Union Seventh Framework funded large-scale cooperative project EUTrigTreat. The rationale, objectives and design of the study including statistical plan and sample size calculations have been published previously. In brief, the study enrolled a contemporary implantable cardioverter defibrillator cohort to test multiple carefully selected risk markers of clinical relevance for prediction of mortality and arrhythmias. The large majority of patients underwent non-invasive programmed ventricular stimulation via their implanted ICDs. Inducibility of sustained ventricular arrhythmia was defined as induction of a single monomorphic ventricular tachycardia lasting for 30 seconds or two polymorphic ventricular tachycardia/ventricular fibrillation episodes requiring cardioversion. A 24-h Holter monitoring was performed using standard devices. The primary endpoint was all-cause mortality. First appropriate implantable cardioverter defibrillator shock was selected as a key secondary endpoint. Cox regression analysis was implemented as described. Risk models for shock and mortality were developed using forward selection among a set of known potential risk factors ( Figs. 1 and 2 and Table 1, Table 2, Table 3).

**Table 1:** Results for univariate Cox regression for prediction of mortality (unadjusted and adjusted for base model).

**Variable**	**Patients**	**unadjusted**			**adjusted**		
		***p***	**HR**	**CI**	***p***	**HR**	**CI**
Age (per 10 years)	635	< 0.0001	2.20	1.79-1.2,71			
LVEF (per 5%)	635	< 0.0001	0.74	0.68–0.81			
NYHA > 2	635	< 0.0001	2.65	1.82–3.86			
eGFR (per 30 ml/min)	623	< 0.0001	0.45	0.35–0.58			
Male gender	635	0.0749	1.60	0.93–2.76			
Ischemic vs. non-ischemic	634	0.0330	1.73	1.04–2.22			
Secondary prevention	634	0.0211	0.61	0.40–0.94			
History of AF	622	< 0.0001	4.02	2.56–6.31			
COPD	635	0.0001	2.78	1.75–4.55			
NTproBNP/BNP (per 100 ng/l)	582	0.0016	1.46	1.23–1.73	0.0155	1.46	1.16–1.84
hs-CRP (per 10 mg/dl)	477	0.0013	1.62	1.29–2.05			
ICD chambers (dual vs. CRT; single vs. others)	635	< 0.0001	0.62; 1.99	0.37–1.02; 1.30–3.05	0.0346	0.55; 1.00	0.32–0.91; 0.64–1.56
Intrinsic QRS (per 10 ms)	535	0.0007	1.13	1.05–1.20	0.3650	1.04	0.96–1.12
Intrinsic QT interval (per 10 ms)	535	0.433	1.02	0.98–1.06	0.3960	0.98	0.94–1.03
Intrinsic QTc interval (per 10 ms)	535	0.0362	1.05	1.005–1.10	0.9000	1.00	0.95–1.05
Inducibility on EP testing	616	0.4280	1.21	0.76–1.93	0.9900	1.00	0.61–1.63
MTWA (A rules)	493	0.0125	1.82	1.13–2.93	0.9020	1.03	0.63–1.70
MTWA (B rules)	493	0.0113	1.82	1.14–2.90	0.8240	1.06	0.65–1.72
Holter mean heart rate (per 10 bpm)	634	0.1930	1.14	0.94–1.39	0.0780	1.21	0.98–1.49
Holter PVC/24 h (per 100/24 h)	632	0.6580	1.00	1.00–1.00	0.8640	1.00	1.00–1.00
Holter nsVT/24 h	632	0.1640	0.98	0.94–1.02	0.3710	0.98	0.95–1.03
Holter SDNN (per 10 ms)	470	0.0075	0.92	0.86–0.98	0.7900	0.99	0.92–1.06
Holter RMSSD (per ms)	473	0.6980	0.83	0.31–2.22	0.7450	0.85	0.31–2.32
Holter DC (per ms)	474	0.0022	0.96	0.94–0.98	0.2450	0.98	0.95–1.01
Holter HRT category (TO/TS abnormal)	434	< 0.0001	3.95	2.06–7.57	0.036	2.05	1.00–4.17
Holter HRT onset (%)	434	0.0012	1.18	1.08–1.28	0.074	1.12	1.00–1.25
Holter HRT slope (ms/RR-interval)	434	0.0001	0.88	0.81–0.95	0.282	0.96	0.90–1.04

(Open field = no adjusted value available, AF = atrial fibrillation, CI = confidence interval, COPD = chronic obstructive pulmonary disease, eGFR = estimated glomerular filtration rate, DC = deceleration capacity, HR = hazard ratio, HRT = heart rate turbulence, hs-CRP = high-sensitivity C-reactive protein, ICD = implantable cardioverter defibrillator, EP = electrophysiological, LVEF = left ventricular ejection fraction, MTWA = microvolt T-wave alternans, PVC = premature ventricular contraction, nsVT = non-sustained ventricular tachycardia, NT-pro-BNP = n-terminal-pro-brain natriuretic peptide, NYHA = New York Heart Association functional class, SDNN = standard deviation of RR intervals, RMSSD = mean square root of mean of squared differences between normal-to-normal RR intervals, TO = turbulence onset, TS = turbulence slope).

**Table 2:** Results of univariate Cox regression for prediction of appropriate shock (unadjusted and adjusted for base model).

**Variable**	***n***	**unadjusted**			**adjusted**		
		***p***	**HR**	**CI**	***p***	**HR**	**CI**
Age (per 10 years)	635	0.6970	0.97	0.98–1.01			
LVEF (per 5%)	635	0.0004	0.87	0.80–0.94			
NYHA > 2	635	0.5060	0.86	0.54–1.36			
eGFR (per 30 ml/min)	623	0.0110	0.72	0.55–0.93			
Male gender	635	0.4140	1.24	0.73–2.12			
Secondary prevention	634	0.0051	1.78	1.19–2.66			
Ischemic vs. non-ischemic	633	0.2040	1.30	0.87–1.95			
COPD	635	0.0130	2.29	1.26–4.16			
History of AF	622	0.7640	1.23	0.68–1.82	0.487	1.17	0.75–1.83
NTproBNP/BNP (per 100 ng/l)	582	0.3350	1.20	0.88–1.63	0.895	1.03	0.65–1.64
hs-CRP (per 10 mg/dl)	477	0.6710	0.90	0.53–1.51			
ICD chambers (dual vs. CRT, single vs. other)	635	0.8880	1.12; 1.03	0.71–1.75; 0.60–1.78	0.7590	1.17; 1.17	0.74–1.86; 0.66–2.11
Intrinsic QRS (per 10 ms)	535	0.0306	1.08	1.01–1.15	0.1140	1.06	0.99–1.14
Intrinsic QT (per 10 ms)	535	0.0736	1.04	1.00–1.08	0.1110	1.04	0.99–1.14
Intrinsic QTc (per 10 ms)	535	0.0208	1.06	1.00–1.11	0.0886	1.05	0.99–1.10
EP inducibility	616	0.0009	2.15	1.40–3.30	0.0101	1.84	1.18–2.89
MTWA (A rules)	493	0.0068	1.85	1.18–2.92	0.0592	1.58	0.98–2.56
MTWA (B rules)	493	0.0152	1.73	1.11–2.69	0.1100	1.46	0.92–2.32
Holter mean heart rate (per 10 bpm)	634	0.1990	0.87	0.70–1.08	0.1580	0.85	0.68–1.07
Holter PVCs/24 h (per 100/24 h)	635	0.2880	1.00	1.00–1.00	0.281	1.00	1.00–1.00
Holter nsVT/24 h	635	0.9870	1.00	0.99–1.01	0.9120	1.00	0.98–1.01
Holter SDNN (per 10 ms)	470	0.6850	1.01	0.96–1.07	0.4310	1.03	0.96–1.09
Holter RMSSD (per ms)	473	0.9110	1.00	0.99–1.01	0.9240	1.00	0.99–1.01
Holter DC (per ms)	474	0.0896	0.97	0.95–1.00	0.2140	0.98	0.95–1.01
Holter HRT category (TO or TS abnormal, TO/TS abnormal)	434	0.2610	1.52; 1.49	0.87–2.64; 0.78–2.85	0.2580	1.60; 1.60	0.87–2.93; 0.78–3.30
Holter HRT onset (%)	434	0.4470	1.04	0.94–1.16	0.7230	1.02	0.91–1.15
Holter HRT slope (ms/RR-interval)	434	0.2640	0.97	0.92–1.02	0.3790	0.97	0.91–1.04

(Open field = no adjusted value available, AF = atrial fibrillation, CI = confidence interval, COPD = chronic obstructive pulmonary disease, eGFR = estimated glomerular filtration rate, DC = deceleration capacity, HR = hazard ratio, HRT = heart rate turbulence, hs-CRP = high-sensitivity C-reactive protein, ICD = implantable cardioverter defibrillator, EP = electrophysiological, LVEF = left ventricular ejection fraction, MTWA = microvolt T-wave alternans, PVC = premature ventricular contraction, nsVT = non-sustained ventricular tachycardia, NT-pro-BNP = n-terminal-pro-brain natriuretic peptide, NYHA = New York Heart Association functional class, SDNN = standard deviation of RR intervals, RMSSD = mean square root of mean of squared differences between normal-to-normal RR intervals, TO = turbulence onset, TS = turbulence slope).

**Table 3:** Risk scores for risk of all-cause mortality and risk of appropriate ICD shock.

Mortality score:
0.0547 × age − 0.0452 × lvef + 0.548 * nyha – 0.0117 * egfr + 0.527 × afib + 0.0000376 × ntprobnp

*Shock score:*
**− 0.0268 × lvef − 0.00883 × egfr + 0.684 × prevention + 0.619 × inducibility**

age = age in years; lvef = left ventricular ejection fraction in %; egfr = estimated glomerular filtration rate in ml/min; afib = (1 if present in history, 0 if absent); ntprobnp = NT-pro BNP in ng/l; prevention = (1 if secondary prevention indication, 0 if primary prevention indication); inducibility = (1 if inducible arrhythmia in electrophysiologic study, 0 if arrhythmia not inducible)
