# Supplementary material for: Data on differential multivariable risk prediction of appropriate shock vs. competing mortality
Source: Data Brief. 2018 Nov 9;21:2110–6. doi: 10.1016/j.dib.2018.11.025 (PMC6262164; doi:10.1016/j.dib.2018.11.025)
Supplement: Supplementary file 1 — Supplementary material [file mmc1.docx]

Data-in-Brief Bergau et al. 2018

All authors: no conflict of interest declared

Markus Zabel, M.D.

Corresponding author
